# Impact of dietary supplementation with biological zinc, selenium nanoparticles, and their combination on growth, carcass characteristics, blood parameters, and meat quality in broiler chickens

**DOI:** 10.5455/javar.2025.l918

**Published:** 2025-06-02

**Authors:** Reem Hasaballah Alhasani

**Affiliations:** 1 Department of Biology, Faculty of Science, Umm Al-Qura University, Makkah, Saudi Arabia

**Keywords:** Hematological parameters, broiler nutrition, carcass, growth, organic nanoparticles

## Abstract

**Objective:**

This study examined the impacts of feeding zinc and selenium (SeNPs) nanoparticles and their combinations (ZnNPs and SeNPs and ZnNPs + SeNPs) on growth efficiency, the carcass, blood indicators, and meat criteria traits in chickens during 38 days.

**Materials and Methods:**

Two hundred forty (Cobb 500) 7-day-old chicks were divided in entirely random form into 4 groups, each group divided into 6 replicates of 10 chicks. Dietary supplements were given in accordance with a corn-soybean diet in the following 4 test treatments: 0.0 (T0), 1.5 mg/kg SeNPs (T1), 2.0 mg/kg ZnNPs (T2), and 1.5 mg/kg SeNPs + 2.0 mg/kg ZnNPs (T3). Water and feed were provided at all times during the 38-day period.

**Results:**

The findings concluded that the mixing of SeNPs, ZnNPs, or its combination as feed addition improved rates of growth, as illustrated by higher “body weight” and reduced “feed intake and feed conversion ratio”. The results indicated that there were no appreciable variations (*p* ≤ 0.05) in carcass measurements between the treatments. Additionally, hematological markers showed significant improvements, with decreased amounts of “uric acid, creatinine, aspartate aminotransferase, alanine aminotransferase, total cholesterol, and low-density lipoprotein”, while high-density lipoprotein values increased in chicks feeding ZnNPs or SeNPs compared to the control group in eating. Additionally, T3 levels were lowered and T4 levels were raised when SeNPs, ZnNPs, or a mixture of the two were given. Additionally, these treatments affected immunological responses, leading to increased immunoglobulin (IgM and IgG) levels. By increasing moisture quantity while maintaining carcass texture, aroma, tenderness, juiciness, and acceptability scores, these supplements also had an impact on meat quality.

**Conclusion:**

The addition of SeNPs and ZnNPs to the chicken diet enhanced immune system function, growth, and blood criteria. This raises the possibility of a substitute for popular growth boosters and organic immune modulators.

## Introduction

Feed constitutes about 60%–70% of poultry production costs, so researchers have turned to feed additives that enhance feed use efficiency, improve production, and reduce costs [1–3]. Recently, organic dietary additives have supplanted chemical growth hormones and antibiotics to accelerate growth and enhance the productivity of avian and cattle [4–7].

Selenium is an essential trace mineral having antiviral and antibacterial effects. It markedly improves cellular tissue stability and production efficiency [8, 9]. Furthermore, Ahmadi et al. [10] reported that regulating hens‘ immune systems and antioxidant levels yielded promising results. Nanomaterials, utilized as medications and feed supplements, consist of minuscule particles with extensive surface area, are non-toxic, exhibit enhanced efficacy, possess numerous surface-active sites, and may be beneficial for biological applications [8].

Medicines used for loading can enhance their bio-availability, resulting in improved absorption of readily transported substances [11]. Many selenium-binding proteins, such as “glutathione peroxidase and thioredoxin reductases”, depend on selenium nanoparticles (SeNPs) for their function, where these compounds help to fight free radicals made during oxidative stress [12, 13].

An important microelement that influences numerous avian physiological processes is zinc, like their “immune systems, hormone production, DNA synthesis, digestion of fats, carbohydrates, and proteins, and antioxidant activity“ [14, 15]. Recent research has utilized ZnNPs as a dietary supplement because of their advantageous effects on avian metabolism and wellness. The antimicrobial properties of ZnNPs enhance the body‘s resistance [16, 17].

Zinc nanoparticle (ZnNP) additives improve “body weight gain (BWG), feed conversion ratio (FCR), meat criteria, and egg production,” as shown by Abd El-Hack et al. [18]. Nutrition has a crucial role in the growth and health of chickens, yet the impact of certain nutrients and trace elements on specific features remains unclear [19]. Moreover, an imbalance of trace minerals increases the likelihood of public health disorders [20].

Our recent study is novel because it thoroughly examines the effects of ZnNPs, SeNPs, and their combination (ZnNPs + SeNPs) as feed additives in broilers over a 38-day period. We pay particular attention to growth performance as well as a variety of physiological and meat quality parameters. Although earlier research has examined the effects of supplementing with selenium or zinc in their individual forms, usually in inorganic or organic forms, our study specifically used these minerals in nanoparticle form, which offers better bioavailability, lower dose requirements, and possibly less toxicity than conventional sources. Our study is novel in its application because the transition to nanotechnology in poultry nutrition is a comparatively unexplored field.

Additionally, by including a combined treatment (ZnNPs + SeNPs), which is less frequently studied in broiler research, we were able to look at possible synergistic impacts between these two trace elements in nanoparticle form.

The purpose of the study was to assess how organic ZnNPs, SeNPs, and their mixes affected the meat criteria, blood signs, carcass characteristics, and growth efficacy of broilers up until the age of 38 days.

## Materials and Methods

### Ethical approval

The Institutional Ethics Committee of the Department of Biology, Faculty of Science, Umm Al-Qura University, Makkah, Saudi Arabia, gave its clearance for this study to be carried out. The procedures outlined in Umm Al-Qura University‘s Animal Care manual were adhered to. The experiment was carried out in compliance with the ARRIVE criteria (https://arriveguidelines.org), and the chicks were cared for in compliance with established and specified norms and regulations.

### Trial design and birds’ management

In March and April of 2023, 7-day-old broilers (Cobb 500) were used in this investigation. The experiment lasted until the chicks were 38 days old. Six replicates of ten chicks each were created from 240 chicks that were split up into four treatments. Every bird was brought up in the same climate. Diets were created for two time periods: 7–21 days for starters and 22–38 days for finishers. Table 1 shows the diet as it was designed according to the requirements of broiler chickens, predicated on NRC [21].

**Table 1.** Composition and chemical analysis of basal diets as fed.

Items	Basal diets	
	Starter (7–21 days)	Finisher (22–38 days)
Ingredients (gm/kg diet)		
Yellow corn	65.00	61.00
Soybean meal 44%	17.00	15.00
Concentrate 45%	12.40	11.50
Dicalcium phosphate	3.00	3.00
Limestone	3.00	3.00
DL-Methionine	0.50	0.20
L-Lysine HCl	1.30	1.00
Soybean oil	10.00	28.50
Total	100	100
Calculated analysis**:		
Dry matter %	91.74	90.43
Crude protein %	23.00	21.00
Metabolizable energy kcal/Kg diet	12.35	12.97
Calcium %	1.00	0.90
Phosphorous (available) %	0.45	0.40
Lysine %	1.20	1.05
Methionine+ cysteine %	0.83	0.74
Crude fiber %	3.56	3.31

* Protein concentrate (45%) its chemical analysis: crude protein: 45%, ME: 2470 kcal/kg diet, calcium: 6.13%, phosphorus: 2.32%, lysine: 2.67%, methionine + cystine: 2.19%, and fiber: 2.18%.

** Calculated according to NRC 1994.

Water and food were continuously available during the trial, which spanned an age range of 7 to 38 days. The following is the base diet with dietary additives for the four trial diets: “0.0 (T0), 1.5 mg/kg SeNPs (T1), 2.0 mg/kg ZnNPs (T2), and 1.5 mg/kg SeNPs + 2.0 mg/kg ZnNPs (T3), respectively.“ For the first, second, and last weeks of the experiment, the ambient temperature was kept at 33°C, 30°C, and 26°C, respectively. The study‘s relative humidity was maintained between 50% and 60%. We also maintained a regular table for them, with 23 h of light and 1 h of darkness. The birds were regularly vaccinated against Newcastle disease (ND), Gumboro, infectious disease bronchitis (IB), and avian influenza (AI) in the following ways: IB and ND via eye drop at 7 days of age, ND and AI via subcutaneous injection at 8 days of age, Gumboro via eye drop at 14 days of age, and IB and ND via drinking water at 18 days of age.

### Studied traits

#### Growth efficiency

FI and body weight were evaluated at 7, 21, and 38 days, along with BWG and FCR at the periods of 7–21, 22–38, and 7–38 days. The following formula was used to calculate the performance index (PI).

#### Carcass Traits

Six hens were chosen from each treatment at the conclusion of the trial. Before being killed, the chosen hens fasted for the entire night. Prior to being killed, the hens were weighed. The inedible parts were taken out, and the feathers that were left were mechanically de-feathered. The percentages of feathers, belly fat, and blood were then determined by weighing the heart, liver, Fabricius gland, and spleen.

### Blood Biochemical and Immune Criteria

Following the experimental conclusion, the birds were killed, and blood samples were taken from each treatment group. To measure different hematological parameters, each sample was separated into tubes that were heparinized and those that were not. The following were measured: “uric acid, creatinine, alanine aminotransferase (ALT), aspartate aminotransferase (AST), high-density lipoprotein (HDL), low-density lipoprotein (LDL), and total cholesterol. Additionally, colorimetric detection of serum immunoglobulins (IgG and IgA)” was performed utilizing commercial kits.

### Meat quality

The broiler‘s major pectoral muscle was used to measure the pH and visually assess the meat‘s quality. Samples of the pectoralis major muscle of birds were kept at 4°C for a full day. A pH meter was employed to measure the pH values by penetrating the muscle; Selim et al. [22] assert that a minimum depth of 1 cm should be utilized following the slicing of the muscles. The meat specimens were examined utilizing a variety of AOAC procedures [23]: drying in the oven (method number 930.15), incineration (method number 942.05), crude protein determination via Kjeldahl (technique number 984.13), and fat determination via Soxhlet (approach number 920.39) for ether extract. A five-point scoring system was used to assess the specimens‘ texture, color, and scent to determine the general embrace of chicken flesh. Color discoloration (5, no discoloration; 1, severe discoloration), texture (5, firm; 1, excessively soft), and odor (5, highly acceptable; 1, significantly unacceptable/off-odor) were among the numerous sensory qualities that the panelists assessed. The meaning of these evaluations is widespread approval (5, highly favorable; 1, extremely unfavorable) [24]. Before being measured, the breast pieces were kept for 24 h at 4°C. The weight difference before and after cooling was divided to determine the drip loss percentage.

### Statistical Analysis

Regression analysis was utilized to assess the impacts of dietary interventions in this fully randomized investigation, and the findings were analyzed using SPSS version 27.0. To validate a quadratic response and create a linear relationship, regression analysis necessitates at least three levels; therefore, the four treatment levels (control, 1.5 mg/kg SeNPs, 2.0 mg/kg ZnNPs, and 1.5 mg/kg SeNPs + 2.0 mg/kg ZnNPs) were considered adequate for this method. Both linear and quadratic regression models were fitted using a general linear model, and the findings were displayed as means ± SE. Tukey‘s post hoc test for multiple comparisons was utilized to estimate variation between projected means to compare treatment effects after regression. Regression analysis was performed utilizing the next statistical model:

Yij = ß0 + ß1 Xi + ß2 Xi^2^ + eij

where eij is the random error, β0 is the intercept (overall mean), β1Xi is the linear impact of the group level (Xi, coded as 0, 1.5, 2.0, or a combination), β2Xi² is the quadratic effect, and Yij is the response variable (e.g., BW, FCR, or blood parameters). To identify statistically significant effects or correlations, a significance criterion of *p* < 0.05 was applied.

## Results

### Growth Performance

Table 2 shows the role of “SeNPs, ZnNPs,“ and the mixture of them on body weight (BW), BWG, feed intake (FI), PI, and FCR. The body weight traits of seven-day-old chickens appeared to have no significant differences among treatments, recommending that the experiment be distributed at random. On day 21, the BW was higher in the untreated group and SeNPs 1.5 mg/kg food than in the other treatments; however, by the end of the trial, the BW was higher in the additional treatments, particularly SeNPs. According to the experimental findings, birds given meals enriched with ZnNPs and SeNPs had reduced FI compared to the untreated group, particularly between days 7 and 21 and over the entire 7–38 experiment period. Additionally, these supplements had an impact on BWG throughout the experiment, with birds fed SeNPs having the highest BWG, followed by chickens fed SeNPs and ZnNPs.

**Table 2.** Effect of dietary SeNPs, ZnNPs, and their combination treatments on broiler growth performance.

Traits	Age (days)	Treatments			SEM		*p* value^1^		
		T_0_	T_1_	T_2_	T_3_		T	L	Q
BW (gm/ bird)	7	132.16	132.50	132.33	132.33	0.09	0.728	0.724	0.438
	21	774.44^a^	716.66^a^	641.66^b^	638.33^b^	19.32	0.005	0.001	0.237
	38	1916.66^b^	2065.00^a^	1816.66^b^	1866.66^b^	31.31	0.004	0.027	0.173
FI (gm/ day)	7–21	65.83^a^	61.18^b^	58.09^b^	60.95^b^	1.01	0.024	0.020	0.025
	22–38	124.27	123.09	111.91	114.37	2.63	0.265	0.099	0.720
	7–38	95.05^a^	92.14^ab^	85.00^b^	87.66^b^	1.61	0.094	0.035	0.313
BWG (gm/ bird/ day)	7–21	45.87^a^	41.72^a^	36.38^b^	36.14^b^	1.38	0.005	0.001	0.236
	22–38	63.45^b^	74.91^a^	65.27^b^	68.24^ab^	1.65	0.038	0.665	0.108
	7–38	57.56^b^	62.34^a^	54.33^b^	55.94^b^	1.00	0.004	0.026	0.174
FCR (gm feed/ gm gain)	7–21	1.43^b^	1.47^b^	1.59^ab^	1.69^a^	0.03	0.029	0.005	0.563
	22–38	1.96	1.65	1.72	1.68	0.05	0.157	0.104	0.188
	7–38	1.65	1.48	1.56	1.57	0.02	0.225	0.526	0.133
PI	38	116.15^b^	140.52^a^	116.32^b^	119.23^b^	3.98	0.057	0.592	0.111

T_0_: control (basal diet); T_1_: SeNPs 1.5 mg/kg diet; T_2_, ZnNPs 2.0 mg /kg diet; T_3_, SeNPs 1.5+ ZnNPs 2.0 mg /kg diet; SEM, standard error of means; a,b Means within a row followed by different superscripts are significantly different (*p* ≤ 0.05).

1 T, overall effects of treatments; L, linear effects of increasing treatment levels of broiler; Q, quadratic effects of increasing treatment levels of broiler; BW, body weight; BWG, body weight gain; FI, feed intake; FCR, feed conversion ratio; PI, performance index.

However, throughout the course of 7–21 days, the FCR increased more in the untreated group than in any of the other groups. The treatment with SeNPs came next. Despite the lack of significant differences across the treatments, the SeNPs treatment had the highest FCR at the end of the experiment. The selection of birds that received SeNPs had the best PI, according to the results. The group that received both SeNPs and ZnNPs came next.

### Carcass traits

Table 3 describes the influence of nutrition with SeNPs, ZnNPs, and the mixtures of them on the element weights of chickens (such as pre-slaughter, blood, post-bleeding, spleen, heart, featherweight, gizzard, liver, and abdominal fat). For all traits of the carcass, the findings of this research obtained no discernible variations among the treatments. The differences were in preslaughter weight, weight after bleeding, weight after feathering, and carcass weight. The weight of the carcass was higher in the ZnNPs department than in the SeNPs department.

**Table 3.** Impact of dietary SeNPs, ZnNPs, and their combination treatments on carcass traits.

Traits	Treatments			SEM		*p* value^1^		
	T_0_	T_1_	T_2_	T_3_		T	L	Q
Preslaughter W	2167.66^a^	2166.66^a^	2151.66^a^	1953.66^b^	34.22	0.029	0.011	0.080
After bleeding W	2118.33^a^	2105.00^a^	2088.33^a^	1890.00^b^	33.43	0.016	0.006	0.061
Blood weight	58.33	61.66	63.33	63.33	3.27	0.959	0.635	0.831
After feather W	2015.00^a^	2025.00^a^	2000.00^a^	1818.33^b^	31.31	0.025	0.012	0.053
Feathers weight	103.33	80.00	88.33	71.66	5.53	0.221	0.089	0.747
Carcass weight	1551.66^b^	1600.00^a^	1620.00^a^	1443.33^c^	28.01	0.026	0.164	0.035
Liver weight	56.47	55.33	58.23	55.11	2.18	0.969	0.961	0.850
Heart weight	9.55	9.51	7.73	8.18	0.33	0.088	0.036	0.655
Gizzard weight	32.35	34.03	32.30	31.15	0.67	0.567	0.418	0.340
Spleen weight	3.33	2.60	2.92	2.78	0.21	0.730	0.538	0.544
Abdominal fat weight	19.63	19.67	22.71	19.74	1.47	0.888	0.827	0.663

T_0_: control (basal diet); T_1_: SeNPs 1.5 mg/kg diet; T_2_, ZnNPs 2.0 mg /kg diet; T_3_, SeNPs 1.5+ ZnNPs 2.0 mg/kg diet; SEM, Standard error of means; a,b,c Means within a row followed by different superscripts are significantly different (*p* ≤ 0.05).

1 T, overall effects of treatments; L, linear effects of increasing treatment levels of broiler; Q, quadratic effects of increasing treatment levels of broiler; W, weight.

### Blood indicators

The effects of adding SeNPs, ZnNPs, or both to the feed on the biochemistry of young chicken blood are displayed in Table 4. The study found that the birds administered feed additives had lower blood levels of uric acid. Particularly for the ZnNPs department, this effect was demonstrated next by the ZnNPs and SeNPs treatments and, lastly, by the SeNPs treatment. Although birds fed foods containing feed additives had decreased blood creatinine concentrations compared to the group without additives, there were no appreciable differences in creatinine concentration between the groups. The final results of this work reported that the amounts of both ALT and AST were reduced in the sections of birds that fed on diets mixing SeNPs, ZnNPs, or the combination of them compared to the group without addition. Mixing SeNPs and ZnNPs into the feed also increased HDL values and reduced LDL and TC values significantly (*p* < 0.05) compared to the control group.

**Table 4.** Influence of dietary SeNPs, ZnNPs, and their combination treatments on serum biochemical parameters.

Indicators	Treatments				SEM	*p* value^1^		
	T_0_	T_1_	T_2_	T_3_		T	L	Q
Kidney functions (mg/dl)								
Uric acid	5.31^a^	4.9^b^	4.4^b^	4.6^b^	0.03	0.001	0.006	0.082
Creatinine	0.35	0.33	0.30	0.33	0.02	0.910	0.635	0.831
Liver functions (U/L)								
AST	245.0^a^	222.0^b^	208.0^b^	220.0^b^	6.42	0.000	0.029	0.147
ALT	6.1^a^	5.2^b^	4.8^b^	5.2^b^	1.03	0.000	0.134	0.235
Lipid profile								
TC (mg/dl)	134.3^a^	125.4^b^	118.4^b^	126.1^b^	7.17	0.003	0.042	0.355
HDL (mg/dl)	91.2^b^	92.5^ab^	94.3^a^	92.5^ab^	3.01	0.001	0.018	0.132
LDL (mg/dl)	44.6^a^	35.2^b^	29.1^b^	35.3^b^	3.07	0.001	0.038	0.451
Thyroid hormones								
T3 (ng/dl)	2.5^a^	2.3^b^	2.4^ab^	2.33^b^	0.02	0.047	0.051	0.138
T4 (ng/dl)	131.2^b^	133.6^a^	134.6^a^	133.3^a^	0.07	0.048	0.016	0.362
Immune response								
IgG (mg/dl)	957.3^c^	1045.0^b^	1072.0^a^	1044.0^b^	40.76	0.020	0.142	0.041
IgA (mg/dl)	179.0^b^	186.2^a^	198.3^a^	186.3^a^	18.09	0.013	0.008	0.452

T0: control (basal diet); T1: SeNPs 1.5 mg/kg diet; T2, ZnNPs 2.0 mg/kg diet; T3, SeNPs 1.5+ ZnNPs 2.0 mg/kg diet; SEM, standard error of means; a,b,c Means within a row followed by different superscripts are significantly different (p ≤ 0.05).

1T, overall effects of treatments; L, linear effects of increasing treatment levels of broiler; Q, quadratic effects of increasing treatment levels of broiler; TC, total cholesterol; ALT, alanine aminotransferase; AST, aspartate aminotransferase; HDL, high-density lipoprotein; LDL, low-density lipoprotein; T3, triiodothyronine; T4, thyroxine; IgG, IgA, immunoglobulins Isotypes G and A.

The impacts of SeNPs, ZnNPs, or both on the thyroid hormone levels of broiler poultry aged 38 days are shown in Table 4. The T3 and T4 concentrations were considerably altered by dietary treatments, as the data show *p* < 0.05. T3 concentration significantly decreased (*p* < 0.05) in departments with SeNPs, ZnNPs, or the two, although T4 concentration increased relative to the control. Furthermore, the results indicated that the broiler chicks‘ immune responses were impacted by their dietary categories. The amounts of “IgA and IgG“ in the groups that received SeNPs, ZnNPs, or both together showed significant differences (*p* < 0.05). These groups‘ IgA and IgG levels were significantly greater than those of the without-addition group.

### Meat quality

A quality examination of meat is presented in Table 5. The results of the chemical composition analysis indicate that the moisture content of the feed additive treatments was higher than that of the without-addition department. The protein levels in each therapy did not, however, differ significantly (*p* < 0.05). However, as compared to the control, the ZnNP and SeNP treatments displayed higher pH values along with decreased amounts of fat, ash, TVBN, and TBA.

The results revealed that traits of carcass maintenance, its “texture, aroma, tenderness, and juiciness,“ were irrespective of the group applied. Additionally, the overall color and suitability ratings of the birds that consumed diets containing SeNPs, ZnNPs, or combinations of the two were significantly higher than those of the birds that consumed the without-addition group (*p* < 0.05).

**Table 5.** Effect of dietary SeNPs, ZnNPs, and their combination treatments on meat quality of broilers.

Parameters	Treatments				SEM	*p* value^1^		
	T_0_	T_1_	T_2_	T_3_		T	L	Q
Chemical composition								
Moisture	61.2^b^	66.5^a^	67.8^a^	66.5^a^	0.71	0.001	0.126	0.051
Protein	20.22	20.9	21.8	20.9	0.52	0.590	0.335	0.731
Fat	13.6^a^	10.3^b^	7.8^c^	10.4^b^	0.07	0.032	0.069	0.147
Ash	0.88^a^	0.41^b^	0.35^b^	0.42^b^	0.08	0.001	0.064	0.035
pH	5.7^b^	6.1^a^	6.5^a^	6.2^a^	0.13	0.040	0.036	0.255
TVBN	6.7^a^	5.2^b^	4.5^c^	5.1^b^	0.17	0.001	0.018	0.040
TB^a^	0.75^a^	0.49^b^	0.31^c^	0.47^b^	0.04	0.001	0.008	0.144
Sensory properties								
Juiciness	9.0^a^	8.7^b^	9.0^a^	8.5^b^	0.12	0.021	0.023	0.082
Tenderness	8.8^a^	8.5^b^	8.8^a^	8.6^b^	0.09	0.035	0.081	0.163
Arom^a^	8.4^a^	8.2a^b^	8.3a^b^	8.4^a^	0.06	0.026	0.147	0.073
Taste	8.7^a^	8.4^b^	8.8^a^	8.6a^b^	0.04	0.023	0.057	0.196
Color properties								
L^*^	60.5^b^	61.1^a^	61.9^a^	61.2^a^	0.51	0.039	0.056	0.087
a^*^	5.9^a^	5.1^b^	5.3^b^	5.0^b^	0.13	0.044	0.073	0.213
b^*^	14.6^b^	15.2^a^	15.6^a^	15.3^b^	0.24	0.041	0.032	0.371

T_0_: control (basal diet); T_1_: SeNPs 1.5 mg/kg diet; T_2_, ZnNPs 2.0 mg/kg diet; T_3_, SeNPs 1.5+ ZnNPs 2.0 mg/kg diet; SEM, standard error of means; a,b,c Means within a row followed by different superscripts are significantly different (p ≤ 0.05).

1 T, overall effects of treatments; L, linear effects of increasing treatment levels of broiler; Q, quadratic effects of increasing treatment levels of broiler; TVBN, total volatile basic nitrogen; TBA, thiobarbituric acid; L*, lightness; a*, redness; b*, yellowness.

## Discussion

ZnNPs and SeNPs can destroy germs and shield cells from harm since they are tiny and have phenolic compounds on their surfaces. These active ingredients—also referred to as phenolic substances—help birds grow and improve their meat quality, blood parameters, and carcass criteria. The study‘s findings demonstrated that incorporating ZnNPs and SeNPs into broiler chicken feed enhanced growth metrics. Our findings are similar to those in the study by Khan et al. [8], showing that chicks do better when SeNPs are incorporated into their diet, where selenium is an important trace element that has antimicrobial and antiviral properties. It markedly improves tissue cell composition and efficacy of production. Furthermore, it has demonstrated encouraging outcomes in modulating the immunological and antioxidant levels of hens [10]. On the other hand, commercial chicken diets usually include little to no zinc and selenium, which may be harmful to the birds. Transforming dietary selenium and zinc into nanoparticles and employing a carrier can enhance their bioavailability [25]. Utilizing minerals in their nanoparticle form as dietary additives in poultry feed has been shown to boost productivity and enhance wellness [9, 26]. Our findings agree with Mahmoud et al. [27], who found that ZnNPs significantly improved BWG and FCR in chickens. Additionally, Fathi et al. [28] showed that chickens fed diets including ZnNPs showed increased body weight and lower feed conversion ratios compared to the control group. Additional research indicates that the incorporation of zinc into broiler diets enhances productivity and feed efficacy [29]. The antimicrobial and immunoregulatory properties of ZnNPs may enhance bird health [15].

The experimental results indicated that there were no appreciable variations in carcass criterion between sections, with the SeNPs and ZnNPs sections having the best carcass weight. Our results align with those of Hattab et al. [14], who discovered that broiler feeds enhanced with ZnNPs significantly improved the yield of the carcass (*p* ≤ 0.05). According to Abd El-Hack et al. [18], the additions of ZnNPs to the diet substantially enhanced hatching yield and blood and feather %, but did not affect other carcass traits. Abd El-Hack et al. [9] demonstrated that SeNPs incorporated into broiler meals improved the production of carcasses and dressing; however, it did not significantly influence other carcass attributes. The NPs‘ shape encourages the liver to retain Se, which raises the carcass yield, dressing ratio, and weight. Furthermore, Ahmadi et al. [10] found that chickens fed sodium selenite kept more selenium in their livers than hens fed selenium-containing organic yeast.

Researchers utilize blood biochemical indicators to evaluate the physical and physiological condition of animals [6, 30]. Therefore, this study looked at these parameters and found that birds that ate meals containing SeNPs and ZnNPs had lower levels of “uric acid, creatinine, AST, ALT, TC, and LDL“ while displaying substantial amounts of HDL. Our findings are similar to those of Abd El-Hack et al. [9,18], who observed that adding SeNPs and ZnNPs to the diets of broilers greatly lowered LDL, TC, and uric acid. It also raised the HDL amount. In addition, Mahmoud et al. [27] reported that incorporating ZnNPs into feed significantly reduced uric acid and TG levels and increased HDL values in chickens. Additionally, Saleh et al. [31] reported that feeding broiler chickens with selenium had a significant positive effect on plasma TG and TC levels. Khan et al. [32] also noted an enhancement in blood biochemical parameters following the addition of organic Se to poultry diets.

The favorable effects of supplementing with SeNPs and ZnNPs are demonstrated by the measured amounts of TC, LDL, and HDL in this study. A byproduct of lipid metabolism, cholesterol is produced in the liver. An elevation in cholesterol levels is linked to hormonal and metabolic disorders, hepatic illness, and renal dysfunction [33]. The significantly lower cholesterol levels linked to the addition of SeNPs and ZnNPs compared to the control diet supported the theory that chickens fed NPs have lower cholesterol levels because their livers‘ lipogenic enzymes are not working as hard. Moreover, the liver, which regulates metabolic, digestive, and productive processes, can be harmed by elevated enzyme amounts. These imbalances can make the body‘s functions worse, leading to worse health and productivity [34]. AST and ALT activities in the bloodstream show how well the liver is working and if it is hurt. High levels of these enzymes are linked to damage to the liver or muscles that happens when the body reacts to stress [35]. AST and ALT levels in the control and test groups differed, according to the current findings. SeNPs‘ and ZnNPs‘ inclusion results in lower AST and ALT levels, which suggests improved liver function.

The immune system of poultry, particularly commercially raised ones, is crucial for their survival and immunity but can be compromised by stressors and environmental stimuli [36]. According to the study‘s findings, broiler chicks‘ T3 and T4 values were considerably changed by SeNPs and ZnNP additives to the diet, with T3 concentrations falling and T4 concentrations rising when SeNPs, ZnNPs, or both were added. These test groups also influenced immune responses, with elevated IgM and IgG values. Nanominerals, including SeNPs, can enhance immunological parameters and bolster resistance to illness [37]. This study suggests a practical way to use SeNPs and ZnNPs to boost chicken immunity. The improved absorption of SeNPs and ZnNPs may be the cause of these results. The current data are like those from Cai et al. [38], who found that adding SeNPs to broiler chicks‘ food increased their humoral immunity by raising the levels of IgG and IgM. Supplementation of dietary SeNPs demonstrated immunostimulatory effects in broiler chicks [39]. The higher levels of serum immunoglobulin may be due to SeNPs‘ important biological role in increasing the number of T-helper cells and encouraging the production of cytokines [40].

The study discovered that while there were no appreciable differences in the protein content, treatments with SeNPs, ZnNPs, or both raised the carcasses‘ moisture content relative to the control. Birds on these diets had better color and acceptability ratings, and the carcass maintained its texture, fragrance, tenderness, and juiciness. This outcome matches with the study of Abd El-Hack et al. [18], which demonstrated that ZnNP components improved fat and moisture content while preserving the sensory attributes of the carcass. Moreover, the pH of the examined birds ranged from 6.1 to 6.2, falling within the standard range of 5.3 to 6.5 [41]. The pH of SeNPs was 5.1, ZnNPs was 5.3, and the group of controls was 5.9.

Furthermore, Salim et al. [22] discovered that ZnNPs lowered the pH in broiler muscle without influencing “color, texture, aroma,“ or overall appeal. Mancini and Hunt [42] indicate that the instrumental measures of L* and a* are readily applicable to muscle color, while the colors denoted by b* (blue and yellow) are not typically associated with meat. Karaoglu et al. [43] indicate that a rise in a* and b* values corresponds with a decrease in the L* value, resulting in a gradual darkening of color. Additionally, muscle redox status and pigment content both affect a* value fluctuation. However, the redox state is the only factor influencing b* values. Additionally, there is a slight link between hemoglobin and methemoglobin amounts and L* measurements. In the current investigation, a* values in muscle were significantly lower due to reduced pigmentation compared to those in the thigh [44]. Bianchi et al. [45] indicated that comparing absolute color values across many studies is challenging due to discrepancies in different color measurements and variations in measuring settings.

Huang et al. [46] showed that administering SeNPs to broilers can mitigate significant muscle deterioration. Wei et al. [47] assert that the incorporation of selenium into broiler feed enhances meat quality, extends preservation duration, and provides economic benefits for broiler products in conjunction with other chemicals. Mahamoud et al. [27] asserted that chickens consuming sodium selenite can significantly reduce cooking loss, shear force, and drip waste. The pH as well as the L* and b* measurements for broiler muscle color can be significantly raised by yeast selenium. Giamouri et al. [48] also found that adding SeNPs to food increases the oxidative stability and Se content of breast meat.

In addition to their impacts on growth, blood parameters, and meat quality, the addition of ZnNPs and SeNPs to broiler diets was assessed for its possible impact on important cell signaling pathways that are essential to cellular function and health. The results of this study were probably influenced by the fact that zinc and selenium, as necessary trace elements, are known to alter a number of signaling cascades that control oxidative stress, immunological responses, and metabolic activities. By functioning as a cofactor and modulator in pathways such as the nuclear factor-kappa B (NF-κB) pathway, which controls inflammation and immunological responses, zinc plays a crucial role in cell signaling. ZnNPs‘ increased bioavailability may help regulate cellular redox states by preventing NF-κB activation under oxidative stress and lowering the generation of pro-inflammatory cytokines such as TNF-α and IL-6. The higher IgM and IgG levels in our study, which indicate an improved humoral immune response, may be explained by this. Zinc also affects the p38 and ERK subfamilies of the mitogen-activated protein kinase (MAPK) pathway, which are implicated in stress adaptation and cell proliferation. Higher body weight and enhanced meat quality indicators, including tenderness and moisture content, suggest that ZnNPs supported MAPK signaling, which in turn likely boosted growth rates and tissue integrity [49, 50].

Selenium works through the Nrf2 (nuclear factor erythroid 2-related factor 2) pathway, a master regulator of antioxidant defenses, mainly by its incorporation into selenoproteins such as glutathione peroxidase (GPx). SeNPs may increase Nrf2 translocation to the nucleus, which would decrease oxidative damage and increase the expression of antioxidant genes (GPx, SOD). This is consistent with our data‘s lower AST, ALT, and LDL values, which show better lipid metabolism and liver function. Additionally, selenium affects the PI3K/Akt pathway, which supports protein synthesis and cell viability. The higher HDL levels and lower FCR, which indicate better nutritional use and metabolic efficiency, may be due to enhanced Akt activation [51, 52].

By focusing on these complementary pathways at the same time, ZnNPs and SeNPs (T3) may have synergistic effects. For example, Se increases Nrf2 and PI3K/Akt to reduce oxidative stress and maintain cellular homeostasis, whereas Zn regulates NF-κB and MAPK to strengthen immunity and growth. The observed systemic advantages, including decreased T3 and increased T4 levels, may be amplified by this dual action, indicating a regulatory influence on thyroid hormone metabolism that may be mediated by zinc-dependent transcription factors and selenoprotein deiodinases [53]. Lastly, ZnNPs and SeNPs‘ positive effects in this study—better development, hematological parameters, immunity, and meat quality—are probably due to their involvement in important signaling pathways such as PI3K/Akt, Nrf2, MAPK, and NF-κB. When compared to conventional mineral forms, these molecular mechanisms provide a new dimension to our findings and demonstrate the possibility of supplementation with nanoparticles as a targeted technique to improve broiler productivity and health.

## Conclusion

According to the study‘s findings, broiler chicks that were given SeNPs, ZnNPs, or a combination of the two had much more efficient growth, as seen by greater BW, BWG, FI, and FCR. The supplement improved the broilers‘ cardiovascular health by lowering LDL and increasing HDL, which had a good effect on blood lipid profiles. The dietary supplement improved thyroid hormones and raised IgG and IgA levels, which strengthened the immune system. The results suggest that ZnNPs and SeNPs may enhance poultry production and immunity. While maintaining the meat‘s acceptability ratings for texture, flavor, tenderness, juiciness, color, and tenderness, the SeNPs or ZnNPs treatments also raised the meat‘s moisture level.

## List of abbreviations

a*, redness

ALT, alanine aminotransferase

AST, aspartate aminotransferase

b*, yellowness

BW, body weight

BWG, body weight gain

FCR, feed conversion ratio

FI, feed intake

HDL, high-density lipoprotein

IgG, IgA, immunoglobulins Isotypes G and A

L*, lightness

LDL, low-density lipoprotein

SeNPs, selenium nanoparticles

T3, triiodothyronine

T4, thyroxine

TBA, thiobarbituric acid

TVBN, total volatile basic nitrogen

ZnNPs, zinc nanoparticles.
